# Comment on Martire et al. Prognostic Role of High-Sensitivity C-Reactive Protein/Albumin Ratio in Heart Failure Patients. *Biomedicines* 2026, *14*, 748

**DOI:** 10.3390/biomedicines14061281

**Published:** 2026-06-04

**Authors:** Samet Yavuz

**Affiliations:** Department of Cardiology, Babaeski State Hospital, 39200 Kırklareli, Türkiye; dr.sametyavuz@gmail.com; Tel.: +90-536-498-0256

**Keywords:** chronic heart failure, hs-CRP/albumin ratio, inflammatory biomarkers, MACE, prognosis, risk stratification

We read with great interest the retrospective cohort study by Martire and colleagues [1], published in March 2026 in *Biomedicines*. In a cohort of 500 outpatients with chronic heart failure (CHF), the authors demonstrated that an hs-CRP/SA ratio ≥ 1.19 was independently associated with an approximately 6.5-fold-higher risk of major adverse cardiovascular events (MACEs) over a median follow-up of 5.2 years. By integrating inflammation and nutritional status into a single index, this biomarker represents a simple and accessible tool for risk stratification in CHF. Nevertheless, we wish to highlight several methodological issues that, in our view, should be addressed before these findings can be safely translated into clinical practice.

In the study, hs-CRP and serum albumin levels were measured only once, at baseline. Both parameters are known to be substantially influenced by acute infections, rheumatological flares, changes in hydration status, diuretic use, and episodes of acute decompensation [2,3]. Given the dynamic nature of CHF, a single measurement is unlikely to capture a patient’s cumulative inflammatory and nutritional burden over a 5-year horizon. As the authors themselves acknowledge in the limitations section, assessing longitudinal changes would more accurately reveal the true prognostic value of this biomarker.

Two different cut-off values were used in the study: the population was dichotomised at the median value of 1.19, whereas the ROC analysis identified 1.454 as the optimal cut-off according to the Youden index. The rationale for this discrepancy is not sufficiently explained in the text. It remains unclear which cut-off should be preferred in clinical practice and how patients falling into the “grey zone” between 1.19 and 1.454 should be classified. Furthermore, it is well established that dichotomising a continuous variable at its median tends to overestimate the magnitude of effect [4]. For this reason, placing greater emphasis on the results in which hs-CRP/SA is treated as a continuous variable would be methodologically more rigorous.

Although NT-proBNP values were measured, they were not included in the Cox regression model because of concerns about overfitting. However, NT-proBNP remains the gold-standard prognostic biomarker in CHF [5]. For the hs-CRP/SA ratio to be translated into clinical practice, its incremental discriminative power beyond NT-proBNP must be clearly demonstrated. In this context, analyses such as the Net Reclassification Improvement (NRI) or the Integrated Discrimination Improvement (IDI) would help establish the true added clinical value of this biomarker. In addition, the study did not separately evaluate whether the hs-CRP/SA ratio offers a significant advantage over hs-CRP or serum albumin alone.

A competing-risks approach was not applied in the MACE analysis. Non-cardiovascular mortality was reported at a rate of 1.7 events per 100 patient-years; such events may preclude the occurrence of MACEs in an elderly CHF population. Because standard Cox regression models tend to overestimate the cumulative incidence of the event of interest in the presence of competing events, we suggest that the Fine–Gray subdistribution hazard model would provide more reliable estimates in this setting [6].

The lack of external validation of the 1.19 cut-off value, as acknowledged by the authors themselves, represents an important limitation regarding the generalisability of the findings. In our view, prospective multicentre validation studies involving different ethnic groups, diverse geographical regions, and patients with more advanced disease stages (e.g., NYHA class IV) are needed.

This Comment is not intended to diminish the scientific contribution of the authors’ valuable work. Rather, it aims to highlight methodological considerations that should be addressed as the hs-CRP/SA ratio moves toward clinical application. We look forward to the authors’ clarifications, particularly regarding the comparative analysis with NT-proBNP and the choice of cut-off values. A response from the original authors would enrich the scientific dialogue on this topic and help clarify the true clinical utility of this promising biomarker.

## Data Availability

No new data were created or analysed in this study. Data sharing is not applicable to this article.
